# Pathways to HIV risk and vulnerability among lesbian, gay, bisexual, and transgendered methamphetamine users: a multi-cohort gender-based analysis

**DOI:** 10.1186/1471-2458-11-20

**Published:** 2011-01-07

**Authors:** Brandon DL Marshall, Evan Wood, Jean A Shoveller, Thomas L Patterson, Julio SG Montaner, Thomas Kerr

**Affiliations:** 1 British Columbia Centre for Excellence in HIV/AIDS, St. Paul's Hospital, 608-1081 Burrard Street, Vancouver, BC, V6Z 1Y6, Canada; 2 School of Population and Public Health, University of British Columbia, 2206 East Mall, Vancouver, BC, V6T 1Z3, Canada; 3 Department of Medicine, University of British Columbia, St. Paul's Hospital, 608-1081 Burrard Street, Vancouver, BC, V6Z 1Y6, Canada; 4 Department of Psychiatry, University of California, 9500 Gilman Drive, La Jolla, California, 92093-0680, USA

## Abstract

**Background:**

Methamphetamine (MA) use continues to be a major public health concern in many urban settings. We sought to assess potential relationships between MA use and individual, social, and structural HIV vulnerabilities among sexual minority (lesbian, gay, bisexual or transgendered) drug users.

**Methods:**

Beginning in 2005 and ending in 2008, 2109 drug users were enroled into one of three cohort studies in Vancouver, Canada. We analysed longitudinal data from all self-identified sexual minority participants (*n *= 248). Logistic regression using generalized estimating equations (GEE) was used to examine the independent correlates of MA use over time. All analyses were stratified by biological sex at birth.

**Results:**

At baseline, 104 (7.5%) males and 144 (20.4%) females reported sexual minority status, among whom 64 (62.1%) and 58 (40.3%) reported MA use in the past six months, respectively. Compared to heterosexual participants, sexual minority males (odds ratio [OR] = 3.74, *p *< 0.001) and females (OR = 1.80, *p *= 0.003) were more likely to report recent MA use. In multivariate analysis, MA use among sexual minority males was associated with younger age (adjusted odds ratio [AOR] = 0.93 per year older, *p *= 0.011), Aboriginal ancestry (AOR = 2.59, *p *= 0.019), injection drug use (AOR = 3.98, *p *< 0.001), having a legal order or area restriction (i.e., "no-go zone") impact access to services or influence where drugs are used or purchased (AOR = 4.18, *p *= 0.008), unprotected intercourse (AOR = 1.62, *p *= 0.048), and increased depressive symptoms (AOR = 1.67, *p *= 0.044). Among females, MA use was associated with injection drug use (AOR = 2.49, *p *= 0.002), Downtown South residency (i.e., an area known for drug use) (AOR = 1.60, *p *= 0.047), and unprotected intercourse with sex trade clients (AOR = 2.62, *p *= 0.027).

**Conclusions:**

Methamphetamine use was more prevalent among sexual minority males and females and was associated with different sets of HIV risks and vulnerabilities. Our findings suggest that interventions addressing MA-related harms may need to be informed by more nuanced understandings of the intersection between drug use patterns, social and structural HIV vulnerabilities, and gender/sexual identities. In particular, MA-focused prevention and treatment programs tailored to disenfranchised male and female sexual minority youth are recommended.

## Background

Like many other marginalised groups, lesbian, gay, bisexual, and transgendered (LGBT) populations experience a range of health inequities and vulnerabilities compared to the general population [1]. In addition to the multiple health conditions that disproportionately affect LGBT populations, sexual minorities also experience significant barriers to accessing appropriate care and prevention services [2,3]. Due in part to the historical invisibility of LGBT persons and a reluctance among some communities to consider sexual minorities as a "legitimate" marginalised group, this population continues to be underrepresented in public health research and practice [4].

A number of studies have demonstrated a high prevalence of substance use and dependence among sexual minority groups [5,6]. For example, methamphetamine (MA) use has been well studied among gay, bisexual, and other men who have sex with men (MSM), particularly in relation to increased sexual risk behaviour and HIV transmission [7-9]. Although much less research has been conducted among sexual minority women, several cross sectional studies have demonstrated that lesbian and bisexual-identified females report significantly higher rates of MA use [10,11]. MA use among women who inject drugs (IDU) has also been associated with sexual- and injection-related HIV risk behaviour [12]. These studies and other research imply important gender differences in the typologies of and adverse health outcomes associated with MA use [13]; therefore, gender-based analyses involving sexual minority populations are needed to better inform effective public health approaches and practice.

Although the individual and psychosocial factors that drive HIV risk within the context of MA use are relatively well understood [14-16], research has only begun to elucidate how environmental and structural determinants link MA use with increased HIV vulnerability [17]. In order to most effectively reduce MA-related exposure to HIV risks, several authors have called for the investigation of personal, social, environmental, and structural correlates of MA use and harms [17,18]. The "risk environment" framework, which posits that factors exogenous to the individual intersect to (re)-produce HIV risk and other drug-related harms [19], provides one such conceptual model to guide investigation of the associations between MA use and HIV vulnerabilities operating at various levels of influence.

Using data collected from three large ongoing prospective cohort studies of drug users in Vancouver, Canada, we sought to determine the prevalence of MA use among sexual minority males and females. Furthermore, relying on a risk environment approach, we assessed the relationships between MA use and a range of individual, social, and structural HIV-related vulnerabilities with the aim of indentifying through which pathways MA use may exacerbate exposure to HIV risk.

## Methods

### Study Design

The At Risk Youth Study (ARYS), Vancouver Injection Drug Users Study (VIDUS) and AIDS Care Cohort to Evaluate Access to Survival Services (ACCESS) are open prospective cohorts of drug users in Vancouver, Canada. These studies comprise a larger program of research focused on the study of the initiation and natural history of injection drug use, and are administered by one research centre (i.e., the British Columbia Centre for Excellence in HIV/AIDS). The risk environment framework is utilized as the theoretical foundation from which to examine how a variety of factors within social, physical, and political space interact to (re)-produce HIV and drug-related harm [19]. Recruitment procedures for the three studies are similar, with the primary modes of enrolment being self-referral, word of mouth, and street outreach. Participants of all studies must have resided in the greater Vancouver region and provided informed consent to be eligible. Each study also had specific eligibility criteria that are detailed briefly here. ARYS consists of drug-using street-involved youth; thus, eligibility criteria included being between the age of 14 and 26 and the use of illicit drugs other than or in addition to marijuana in the past 30 days. VIDUS is a study of HIV-negative IDU in which all participants must have injected an illicit drug in the past 6 months to be eligible for inclusion. ACCESS is a cohort of HIV-positive individuals, who, similar to those in ARYS, must have recently used an illicit drug other than or in addition to marijuana. Detailed sampling and recruitment procedures for these three cohorts have been described elsewhere [20-22]. In this analysis, we combined data from all three studies to achieve a sample size with sufficient power to examine MA use among the sub-sample of participants who identified as a sexual minority. While combining data from studies with different inclusion criteria may present some challenges, we note that all studies rely on harmonized recruitment and data collection tools. Furthermore, combining the datasets permitted an examination of MA use patterns across a diverse spectrum of drug users (e.g., street-involved youth, older IDU) in our setting.

At baseline and semi-annually, participants completed a lengthy interviewer-administered questionnaire. Sociodemographic data, as well as information pertaining to drug use patterns, risk behaviours, and health care utilisation are collected. The survey for each study consists of a uniform set of questions, which permits the aggregation and analysis of data from all enrolled participants. Nurses collected blood specimens for HIV and hepatitis C serology and also provided basic medical care and referrals to appropriate health care services. Participants received $20 for each study visit. All studies have been approved by the University of British Columbia/Providence Health Care Research Ethics Board.

### Study Sample

Data from each cohort used in this analysis was collected during the same time frame; thus, all individuals were observed over the same follow-up period. All participants who completed a baseline survey between September 2005 and May 2008 were eligible for inclusion. At baseline, participants were asked to identify their biological sex at birth and their current sexual orientation. "Sexual minority status" was defined as answering affirmatively to one of: gay, lesbian, bisexual, transsexual, transgendered, or other. Participants who refused to report their sex at birth or current sexual and gender identity were excluded from this analysis.

### Study Hypotheses

The primary hypothesis guiding this analysis was based on the risk environment framework and a careful assessment of prior literature investigating the relationship between MA use and HIV risk behaviour. We hypothesized that MA use among sexual minority drug users would be associated with differing exposure to individual, social, and structural HIV vulnerabilities. In an effort to build on previous studies [16,23,24], we sought not only to examine individual-level HIV risk behaviour but also contextual factors including homelessness, neighbourhood of residence, the consumption of drugs in public, and the regulation of these spaces by law enforcement personnel. We also considered the relationship between MA use and physical violence and depression, which have been identified as independent risk factors for HIV infection [9,25]. Finally, we hypothesized that the relationship between MA use and these factors would differ significantly between sexual minority males and females.

### Variables of Interest

The primary outcome of interest was ascertained by examining responses to the questions, "In the last six months, did you use non-injection crystal methamphetamine?" and "In the last six months, did you inject crystal methamphetamine?" Participants who responded "yes" to either or both questions were defined as crystal methamphetamine (MA) users in all subsequent analyses. We also determined the proportion of participants reporting daily or greater use of injection or non-injection MA use in the past 6 months, respectively. All variables examined in this study, including the outcomes and independent variables of interest, were assessed consistently and equivalently across all three studies.

Based on prior literature examining MA use among marginalised populations [12,26-29], we assessed as explanatory variables a broad set of sociodemographic characteristics, drug use variables, sexual activities, markers of violence and depression, and contextual factors. These variables were also chosen to represent both "micro"- (i.e., the immediate social environment of drug use) and "macro"- (i.e., the societal, economic, and legal context that structure drug use and harm) levels articulated by the risk environment framework [19]. Sociodemographic characteristics examined included age (per year older), Aboriginal ancestry (yes versus no), current relationship status (single/dating versus married/regular partner), and baseline HIV status (positive versus negative). All other variables (unless otherwise indicated) referred to behaviours or activities in the past 6 months since the date of the interview. Drug use variables assessed included other stimulant use (i.e., non-injection cocaine use and crack use, respectively), any injection drug use, experiencing a non-fatal overdose, and binge drug use. As defined previously [30], the latter was operationalised as the self-reported use of drugs more often than usual. We also examined the following sexual activities: number of casual or regular partners excluding those in the context of sex work (>1 versus ≤1); any vaginal or anal unprotected intercourse with casual or regular partners (yes versus no); and sex trade work, defined as a categorical variable with "no" as the reference level and consistent condom use with all clients and any unprotected intercourse with clients as the second and third levels, respectively. We ascertained involvement in (i.e., committing) and exposure to (i.e., experiencing) physical violence (yes versus no). We also used the Center for Epidemiologic Studies Depression Scale (CES-D) with a cut-off of ≥16 to measure the level of depressive symptomatology among participants [31]. Finally, contextual factors examined included: residency in the Downtown South (DTS), an area known as a mixed business and entertainment district that is also inhabited by a large street youth population [32]; homelessness (yes versus no); having a warrant or area restriction (i.e., "no go zone") impact access to services or influence where drugs are consumed or purchased (yes versus no); and using drugs in public spaces (>75% of the time versus ≤75% of the time). Warrants and area restrictions are legal orders to restrict access to certain areas of the city, and are commonly issued by law enforcement personnel in an attempt to disrupt crime and reduce street level disorder [33].

### Statistical Analysis

As a preliminary analysis, we compared the baseline sociodemographic characteristics and MA use patterns between heterosexual and sexual minority participants, stratified by biological sex at birth. The Pearson chi-square test was used to compare categorical variables and the Wilcoxon rank sum test was used for continuous variables. We then identified the longitudinal correlates of MA use by using generalized estimating equations (GEE) with a logit link for binary outcomes. GEE were appropriate for this analysis since the factors associated with recent MA use over the baseline and four follow-up periods were serial (i.e., time-dependent) variables. GEE account for the correlation between repeated measures for each subject; thus, valid estimates of association and standard errors are obtained [34]. Since GEE models incorporate periods during which participants report engaging and not engaging in the outcome, data from all baseline and follow-up interviews were used in this analysis.

Since a primary objective of this study was to determine whether the correlates of MA use differed between males and females, we stratified the analyses by biological sex at birth and constructed two multivariate models. We applied a modified backward stepwise procedure to select covariates based on two criteria: the Akaike information criterion (AIC) and type-III *p*-values [35]. Lower AIC values indicate a better overall fit and lower *p*-values indicate higher variable significance. Starting with a full model containing all variables that were significant in bivariate analyses at *p *< 0.10, covariates were removed sequentially in order of decreasing *p*-values. To compensate for potential variations in recruitment and selection procedures between studies, we also adjusted each model for cohort of enrolment. At each step, the *p*-values of each variable and the overall AIC were recorded, with the final model having the lowest AIC. Statistical analysis was conducted using SAS version 9.1.3 (SAS Institute Inc., Cary, North Carolina, USA) and all *p*-values are two-sided.

## Results

### Sample Characteristics

Between September 2005 and May 2008, 2109 unique individuals were enrolled into the ARYS, VIDUS or ACCESS cohorts. A total of 14 (0.7%) refused to report their sex at birth or current sexual/gender identity and were thus excluded for the analysis. Of the 2095 eligible participants, 1389 (66.3%) were male and 706 (33.7%) were female. Among all participants, the median age at baseline was 37.0 (IQR: 24.7 - 45.4) and 641 (30.6%) were of Aboriginal ancestry. The majority identified their sexual or gender identity as heterosexual (*n *= 1847, 88.2%), followed by bisexual (*n *= 168, 8.0%), gay (*n *= 43, 2.1%), lesbian (*n *= 9, 0.4%), and transsexual, transgendered, or other (*n *= 28, 1.3%). Among those who reported their biological sex at birth as female, 144 (20.4%) identified as a sexual minority compared to only 7.5% (n = 104) of biological males.

### Baseline Methamphetamine Use

Sociodemographic characteristics and methamphetamine use patterns for males and females stratified by sexual orientation are displayed in Table 1. At baseline, sexual minority males were more likely to be younger (median = 33 versus 39, *p *= 0.001), HIV positive (40.4% versus 21.2%, *p *< 0.001), and of Aboriginal ancestry (40.4% versus 23.7%, *p *< 0.001). In contrast, sexual minority females were less likely to be of Aboriginal ancestry (33.3% versus 43.9%, *p *= 0.023). Among both males and females, sexual minority participants were significantly more likely to report injection and non-injection MA use in the past 6 months (Table 1). Notably, over half (62.1%) of sexual minority males reported recently using MA, and a significant proportion (16.7%) reported injecting MA at least daily. Approximately half (*n *= 142, 57.3%) of sexual minority participants reported having used MA for at least a year since the date of the baseline interview.

**Table 1:** Baseline sociodemographic characteristics and methamphetamine use patterns among ARYS, VIDUS, and ACCESS participants, stratified by biological sex at birth and self-identified sexual orientation (n, % unless otherwise indicated).

	Male (*N *= 1389)				Female (*N *= 706)			
**Characteristic**	Sexual Minority* (*n *= 104)	**Heterosexual (*n *= 1285)**	**OR (95%CI)**	***p*-value**	Sexual Minority* (*n *= 144)	**Heterosexual (*n *= 562)**	**OR (95%CI)**	***p*-value**

Age (median, IQR)	33 (24 - 42)	39 (25 - 47)	0.97 (0.95 - 0.99)	0.001	31 (23 - 41)	35 (24 - 44)	0.98 (0.97 - 1.00)	0.053
Aboriginal ancestry								
Yes	42 (40.4)	305 (23.7)	2.18 (1.44 - 3.29)	<0.001	48 (33.3)	246 (43.9)	0.64 (0.44 - 0.94)	0.023
No	62 (59.6)	980 (76.3)			96 (66.7)	315 (56.2)		
Relationship status								
Single/dating	73 (70.2)	927 (72.4)	0.90 (0.58 - 1.39)	0.634	90 (62.5)	307 (55.4)	1.34 (0.92 - 1.95)	0.127
Married/regular partner	31 (29.8)	354 (27.6)			54 (37.5)	247 (44.6)		
HIV status								
Positive	42 (40.4)	272 (21.2)	2.52 (1.67 - 3.82)	<0.001	41 (28.5)	159 (28.3)	1.01 (0.67 - 1.51)	0.966
Negative	62 (59.6)	1013 (78.8)			103 (71.5)	403 (71.7)		
Any meth use^†^								
Yes	64 (62.1)	388 (30.5)	3.74 (2.47 - 5.67)	<0.001	58 (40.3)	150 (27.2)	1.80 (1.23 - 2.64)	0.003
No	39 (37.9)	884 (69.5)			86 (59.7)	401 (72.8)		
Any non-injection meth use^†^								
Yes	38 (36.5)	223 (17.5)	2.71 (1.78 - 4.15)	<0.001	36 (25.0)	89 (16.0)	1.75 (1.12 - 2.71)	0.013
No	66 (63.5)	1050 (82.5)			108 (75.0)	466 (84.0)		
Daily non-injection meth use^†^								
Yes	11 (10.6)	39 (3.1)	3.72 (1.84 - 7.50)	<0.001	8 (5.6)	20 (3.7)	1.56 (0.67 - 3.62)	0.296
No	93 (89.4)	1226 (96.9)			135 (94.4)	527 (96.3)		
Any injection meth use^†^								
Yes	43 (41.4)	262 (20.4)	2.75 (1.82 - 4.15)	<0.001	39 (27.1)	100 (18.0)	1.69 (1.10 - 2.59)	0.016
No	61 (58.6)	1021 (79.6)			105 (72.9)	455 (82.0)		
Daily injection meth use^†^								
Yes	17 (16.7)	45 (3.5)	5.45 (3.00 - 9.95)	<0.001	9 (6.4)	16 (2.9)	2.27 (0.98 - 5.24)	0.066
No	85 (83.3)	1229 (96.5)			132 (93.6)	532 (97.1)		

Notes: * "sexual minority" refers to lesbian, gay, bisexual, transgendered, transsexual, or other orientation; † refers to activities in the past 6 months.

### Longitudinal Correlates of Methamphetamine Use

In Table 2, we report the results of the longitudinal analysis examining the factors associated with MA use among sexual minority males. Bivariate analyses indicated that male MA users were more likely to experience a variety of sexual HIV risks and vulnerabilities, including for example multiple recent sex partners (odds ratio [OR] = 1.91, *p *= 0.002), unprotected intercourse (OR = 1.86, *p *= 0.004), and unprotected intercourse in the context of sex work (OR = 3.25, *p *= 0.005). MA using men were also more likely to report injection drug use (OR = 2.31, *p *= 0.004), experience physical violence (OR = 1.76, *p *= 0.004), commit physical violence (OR = 1.90, *p *= 0.025) and exhibit depressive symptoms (OR = 1.79, *p *= 0.010). In multivariate analysis, independent correlates of MA use among sexual minority males included: younger age (adjusted odds ratio [AOR] = 0.93, *p *= 0.011), Aboriginal ancestry (AOR = 2.59, *p *= 0.019), injection drug use (adjusted odds ratio [AOR] = 3.98, *p *< 0.001), unprotected sexual intercourse (AOR = 1.62, *p *= 0.048), increased depressive symptoms (AOR = 1.67, *p *= 0.044), and having an area restriction impact access to services or influence where drugs are used or purchased (AOR = 4.18, *p *= 0.008).

**Table 2:** Longitudinal analysis of factors associated with crystal methamphetamine use† among sexual minority* males (n = 104).

	Bivariate			Multivariate		
**Characteristic**	**Odds Ratio**	**95% CI**	***p*-value**	**Adjusted Odds Ratio**	**95% CI**	***p*-value**

*Sociodemographic Characteristics*						
Age (per year)	0.92	0.89 - 0.96	<0.001	0.93	0.88 - 0.98	0.011
Aboriginal ancestry (yes vs. no)	2.37	1.17 - 4.79	0.016	2.59	1.17 - 5.77	0.019
Relationship status (single/dating vs. married/partner)	0.96	0.65 - 1.42	0.842			
HIV Status (positive vs. negative)	0.50	0.24 - 1.00	0.051			
*Drug Use*						
Non-injection cocaine use^† ^(yes vs. no)	2.44	1.09 - 5.44	0.029			
Crack use^† ^(yes vs. no)	1.47	0.89 - 2.43	0.133			
Any injection drug use^† ^(yes vs. no)	2.31	1.30 - 4.11	0.004	3.98	1.85 - 8.57	<0.001
Overdose^† ^(yes vs. no)	1.52	0.83 - 2.77	0.172			
Binge drug use^† ^(yes vs. no)	1.50	0.90 - 2.50	0.118			
*Sexual Activities*						
Number of sex partners^† ^(>1 vs. ≤1)	1.91	1.28 - 2.86	0.002			
Unprotected intercourse^† ^(yes vs. no)	1.86	1.22 - 2.84	0.004	1.62	1.01 - 2.60	0.048
Sex trade work^† ^(ref = no sex trade work)						
Consistent condom use with clients^† ^(yes vs. ref)	2.79	1.62 - 4.82	<0.001			
Any unprotected sex with clients^† ^(yes vs. ref)	3.25	1.44 - 7.37	0.005			
*Violence & Depression*						
Experience physical violence^† ^(yes vs. no)	1.76	1.20 - 2.59	0.004	1.47	0.93 - 2.32	0.100
Commit physical violence^† ^(yes vs. no)	1.90	1.09 - 3.31	0.025			
Clinical depression (CES-D^‡ ^≥16 vs. <16)	1.79	1.15 - 2.79	0.010	1.67	1.01 - 2.76	0.044
*Contextual Factors*						
Downtown South residency (yes vs. no)	1.45	0.90 - 2.34	0.124			
Homeless^† ^(yes vs. no)	1.76	1.00 - 3.09	0.050			
Area restrictions influence drug use (yes vs. no)	4.02	0.87 - 18.54	0.075	4.18	1.46 - 11.95	0.008
Use drugs in public^† ^(>75% vs. ≤75% of the time)	1.53	0.96 - 2.43	0.073			

Notes: model adjusted for cohort of recruitment; * "sexual minority" refers to lesbian, gay, bisexual, transgendered, transsexual, or other orientation; † refers to activities in the past 6 months; ‡ CES-D refers to the Center for Epidemiologic Studies Depression Scale.

Increased sexual HIV vulnerabilities were also observed among MA-using sexual minority females (Table 3). For example, females reporting recent MA use were more likely to have multiple regular or casual sex partners (OR = 1.55, *p *= 0.029). Several associations that were observed among MA-using males were also significant among females. For example, female MA users were younger (OR = 0.95, *p *= 0.005), more likely to inject drugs (OR = 1.68, *p *= 0.011), and reported elevated rates of unprotected intercourse in the context of sex work (OR = 3.27, *p *= 0.001). In contrast, MA-using females were less likely to be of Aboriginal ancestry (OR = 0.41, *p *= 0.012).

**Table 3:** Longitudinal analysis of factors associated with crystal methamphetamine use† among sexual minority* females (n = 144).

	Bivariate			Multivariate		
**Characteristic**	**Odds Ratio**	**95% CI**	***p*-value**	**Adjusted Odds Ratio**	**95% CI**	***p*-value**

*Sociodemographic Characteristics*						
Age (per year)	0.95	0.92 - 0.99	0.005			
Aboriginal ancestry (yes vs. no)	0.41	0.21 - 0.82	0.012	0.55	0.25 - 1.21	0.137
Relationship (single/dating vs. married/partner)	1.07	0.76 - 1.49	0.708			
HIV Status (positive vs. negative)	0.62	0.90 - 1.30	0.209			
*Drug Use*						
Non-injection cocaine use^† ^(yes vs. no)	1.79	1.06 - 3.04	0.030	1.66	0.94 - 2.92	0.079
Crack use^† ^(yes vs. no)	0.95	0.71 - 1.27	0.730			
Any injection drug use^† ^(yes vs. no)	1.68	1.13 - 2.50	0.011	2.49	1.42 - 4.39	0.002
Overdose^† ^(yes vs. no)	1.47	0.90 - 2.41	0.126			
Binge drug use^† ^(yes vs. no)	1.18	0.77 - 1.81	0.452			
*Sexual Activities*						
Number of sex partners^† ^(>1 vs. ≤1)	1.55	1.05 - 2.30	0.029			
Unprotected intercourse^† ^(yes vs. no)	0.97	0.65 - 1.45	0.897			
Sex trade work^† ^(ref = no sex trade work)						
Consistent condom use with clients^† ^(yes vs. ref)	1.30	0.88 - 1.93	0.189	1.16	0.72 - 1.87	0.543
Any unprotected sex with clients^† ^(yes vs. ref)	3.27	1.60 - 6.68	0.001	2.62	1.12 - 6.14	0.027
*Violence & Depression*						
Experience physical violence^† ^(yes vs. no)	1.24	0.88 - 1.75	0.210			
Commit physical violence^† ^(yes vs. no)	1.12	0.81 - 1.54	0.499			
Clinical depression (CES-D^‡ ^≥16 vs. <16)	0.85	0.66 - 1.09	0.204			
*Contextual Factors*						
Downtown South residency (yes vs. no)	1.45	1.00 - 2.10	0.053	1.60	1.01 - 2.54	0.047
Homeless^† ^(yes vs. no)	1.19	0.86 - 1.64	0.299			
Area restrictions influence drug use (yes vs. no)	0.59	0.28 - 1.23	0.160			
Use drugs in public^† ^(>75% vs. ≤75% of the time)	1.18	0.77 - 1.81	0.446			

Notes: model adjusted for cohort of recruitment; * "sexual minority" refers to lesbian, gay, bisexual, transgendered, transsexual, or other orientation; † refers to activities in the past 6 months; ‡ CES-D refers to the Center for Epidemiologic Studies Depression Scale.

In a multivariate analysis, several unique correlates of MA use emerged among sexual minority females. In contrast to males, MA-using females were more likely to reside in the Downtown South neighbourhood (AOR = 1.60, *p *= 0.047). Furthermore, MA use among sexual minority females was independently associated with unprotected intercourse with sex trade clients (AOR = 2.62, *p *= 0.027). Similar to males, MA-using females were more likely to report injection drug use (AOR = 2.49, *p *= 0.002).

## Discussion

In the current study, we observed a high prevalence of MA use among sexual minority males and females in comparison to heterosexual participants. We also found that, consistent with the risk environment framework, MA use was associated with an array of individual, social, and contextual HIV-related risks and vulnerabilities among sexual minority drug users.

Although some correlates of MA use (e.g., younger age and injection drug use) were significant for both sexes, several important differences were observed. For example, unprotected intercourse involving regular or casual partners was more common among males who reported using methamphetamine, while unprotected intercourse in the context of sex work was associated with MA use among females. Furthermore, only MA-using males were more likely to experience depressive symptoms and report having area restrictions (i.e., "no go" zones) impact access to services of influence where drugs are used or purchased. These findings may be due to the fact that sexual minority males reported heavier MA use patterns compared to females, and thus may be more likely to experience individual (i.e., depressive symptoms) and contextual (i.e., exposure to law enforcement) MA-related sequelae. Finally, Aboriginal ancestry was positively associated with MA use among males but inversely associated with MA use among females.

Consistent with other studies [7,8,36], MA use was linked with unprotected intercourse among sexual minority men. Although we were unable to ascertain the context in which instances of unprotected intercourse occurred, we point to other research indicating that homeless sexual minority males frequently experience sexual victimization and abuse from partners [37]. Although more research is required to fully elucidate casual mechanisms, we hypothesize that the relationship between sexual risk and MA use observed among this sample of street-involved sexual minority men is less a function of desire to enhance sex but is in fact a marker of increased vulnerability within sexual relationships. A similar pathway may also explain the marginal association between MA use and experiencing physical violence observed among males in this study.

In multivariate analysis, among the subsample of females engaging in sex work, MA use was associated with unprotected intercourse with clients. This finding can be situated within a growing literature demonstrating how social and structural inequities hinder the individual agency of drug-using survival sex workers to practice HIV prevention and harm reduction with clients [38]. In a recent study of female sex workers (FSW) in Vancouver, Canada, Shannon et al. [39] demonstrated that MA use is associated with living and working in marginalised public spaces (e.g., industrial areas). These areas have been shown in previous research to be settings of increased risk of violence and pressure from clients to engage in unprotected sex [40]. Our results support this work and indicate that MA use may augment the adverse impact of social-structural factors in the production of HIV risk among sexual minority women involved in survival sex work.

The strongest correlate of MA use among sexual minority men was reporting that a warrant or area restriction impacted access to services or influenced where drugs are consumed or purchased. The socio-legal regulation of public space and its negative impact on the health of homeless people and street-level drug users has been described previously [41]. Recent work also suggests that the displacement of street-involved young people using warrants or area restrictions exacerbates stigma and increases sexual vulnerability and HIV risk [42]. Our findings suggest that having one's movements restricted may also encourage transitions in drug use (including initiation of MA use), due perhaps to the forced removal of drug users from normative environments and social networks. It is also possible that MA users are at an increased risk of incarceration and other interactions with the legal system, and are thus more likely to be affected by punitive policies such as warrants and area restrictions. This form of marginalisation (produced by policies and practices meant to *reduce *exposure to street-level drug use and violence) is one example of a population-level intervention that may *exacerbate *inequity and worsen the health of vulnerable groups [43].

These findings also support the urgent need for increased resources and programming directed towards LGBT people who use methamphetamine. In order to inform more effective interventions to reduce the harms associated with MA, researchers must clearly articulate how social/structural processes impact the health of sexual minorities. Once clearly identified, these factors can then be the target of broad sets of evidence-based interventions to reduce health inequities and improve overall health. For example, changes in government policy along with community mobilization and solidarity programs have been shown to be highly successful at reducing HIV risk among survival sex workers [44]. Programs that support capacity-building in marginalised communities have also been shown to reduce health inequity and improve health outcomes [45]. Although further research is required to elucidate the potential impact of specific enforcement practices (e.g., area restrictions) on MA use and related harms, improved coordination between policing and public health initiatives may represent another opportunity to prevent the (un)-intended consequences of public policies meant to reduce crime and street disorder [46]. Finally, additional research is required to identify specific programmatic needs of subpopulations within sexual minority communities, including for example transgendered youth.

To complement structural interventions, some behavioural approaches (e.g., cognitive behavioural therapy) offer promise [47]. For example, LGBT-specific substance abuse treatment programs have been found to reduce engagement in high-risk sex among drug-using gay men [48]. Harm reduction programs, particularly those offering tailored services for MA users, are effective and well received by clients [49]. Finally, given the associations between Aboriginal ancestry, sexual orientation, and MA use observed in this study, methamphetamine-specific programming should carefully identify the manner in which cultural and sexual identities shape drug use and HIV risk within specific contexts and settings.

This study has a number of limitations that should be noted. The ARYS, VIDUS, and ACCESS cohorts are not random samples of the eligible population; thus, findings may not necessarily be generalizable to other urban areas in which MA use is prevalent. The small sample sizes may have resulted in insufficient power to detect true associations, particularly after adjustment for confounding. Furthermore, data from three studies with different inclusion criteria were combined and analysed, which may have resulted in cohort or selection effects. To mitigate the potential impact of these biases, all sampling and data collection procedures were harmonized, and all multivariate models were adjusted for cohort of recruitment. We note that all behaviours ascertained in this study were self-reported, and we were unable to confirm MA use with urine samples or other measures. We also recognize that our primary analysis was restricted to individuals who self-identified as a sexual minority; therefore, heterosexual-identified individuals who engaged in same-sex activity were excluded. We chose not to rely on behavioural eligibility criteria (e.g., MSM), as we feel, as do others [50], that ignoring sexual identity in HIV prevention efforts obscures the social dimensions of sexuality that are critical for the development of effective and culturally relevant public health interventions. However, we note that public health efforts should be made to provide appropriate services for non-LGBT identifying MSM/WSW, including programs that explicitly acknowledge and accept diverse sexual experiences and identities [51]. We were unable to ascertain motivations for MA use, which if examined may have accounted some of the observed differences in the characteristics and consequences of MA use between male and female participants in this study. Finally, although our data are longitudinal, we do not wish to imply that this analysis provides thorough insight into the causal pathways linking MA use and HIV risk with broader social and structural inequities.

## Conclusion

We have demonstrated in a longitudinal data set a high prevalence of MA use among a cohort of street-involved sexual minority drug users. To our knowledge, this is the first study to extend the risk environment approach as a theoretical foundation from which to understand the contexts of risk associated with MA use among LGBT populations. Consistent with the risk environment framework, MA use was associated with distinct sets of individual, social, and structural HIV risks and vulnerabilities among women and men, respectively; therefore, comprehensive interventions that involve sectors outside of health (e.g., housing, law enforcement), in addition to drug-specific approaches tailored to LGBT populations, are required to reduce HIV vulnerability and MA-related harms. Finally, researchers and public health practitioners must identify multi-sector population-level interventions that do not exacerbate inequity but successfully mitigate health inequities among vulnerable populations.

## Competing interests

Dr Montaner reported receiving educational grants from and serving as an ad hoc advisor to or speaking at various events sponsored by Abbott Laboratories, Agouron Pharmaceuticals Inc, Boehringer Ingelheim Pharmaceuticals Inc, Borean Pharma AS, Bristol-Myers Squibb, DuPont Pharma, Gilead Sciences, GlaxoSmithKline, Hoffmann-La Roche, Immune Response Corporation, Incyte, Janssen-Ortho Inc, Kucera Pharmaceutical Company, Merck Frosst Laboratories, Pfizer Canada Inc, Sanofi Pasteur, Shire Biochem Inc, Tibotec Pharmaceuticals Ltd, and Trimeris Inc. All other authors declare that they have no competing interests.

## Authors' contributions

TK had full access to all of the data and takes responsibility for the integrity of the results and the accuracy of the statistical analysis. BM, TK, and JS conceived the study concept and design, and BM was responsible for the statistical analysis and composition of the manuscript. BM led the interpretation of the results, with significant scientific input from JS, EW, TP, JM, and TK. The manuscript was edited and revised by BM, EW, JS, TP, JM, and TK. All authors read and approved the final version of the manuscript.

## Pre-publication history

The pre-publication history for this paper can be accessed here:

http://www.biomedcentral.com/1471-2458/11/20/prepub
